# COVID-19: Measles and Antibiotic Resistance Are a Matter of Concern

**DOI:** 10.3390/pathogens10040449

**Published:** 2021-04-09

**Authors:** Giovanni Di Guardo

**Affiliations:** General Pathology and Veterinary Pathophysiology, Veterinary Medical Faculty of the University of Teramo, Località Piano d’Accio, 64100 Teramo, Italy; gdiguardo@unite.it

**Keywords:** COVID-19, SARS-CoV-2, measles, measles virus, antibiotic resistance

An interesting Perspective article recently published in the *New England Journal of Medicine* offers an insightful overview on the benefits provided by the mass vaccination of children against Severe Acute Respiratory Syndrome CoronaVirus-2 (SARS-CoV-2), the betacoronavirus responsible for the dramatic CoronaVirus Disease-2019 (COVID-19) pandemic [1].

To this aim, an elegant parallel is also made with measles, a disease still killing 100,000 people globally each year [2], and with many recent outbreaks affecting unvaccinated children [1]. Noteworthily, measles virus (MeV) is responsible—in humans as well as in experimentally infected macaques—for the partial loss of antibody-based immune memory against previously encountered pathogens (either naturally or following vaccination) [2]. Consequently, the occurrence of a MeV infection’s outbreak among COVID-19-vaccinated children and/or adults could have very negative effects, especially when an adequate “herd immunity” level should be reached.

Such an alarming scenario is made even more scary by the SARS-CoV-2 variants (so-called “variants of concern”, VOC) circulating in many Countries, both European (including Italy) and non-European, such as the “English” (alias “B.1.1.7”), “South African” (alias “B.1.351”), “Brazilian” (alias “P.1”), “Nigerian” (alias “B.1.525”) and “Mexican” (alias “B.1.1.222”) VOC, which were preceded by the one known as “cluster 5” (characterized by the “Y453F” mutation within the “receptor-binding domain” of SARS-CoV-2 spike protein), found during late spring and early summer of 2020 in mink farms in the Netherlands and Denmark [3]. Indeed, concern exists that the aforementioned VOC, with special reference to the South African and Brazilian ones, could escape—at least partially—the immunity conferred by a previous infection, as well as by the currently available anti-SARS-CoV-2 vaccines [4,5].

Many comparisons have also been made, in the biomedical literature and elsewhere, between COVID-19 and “Spanish flu”, although no antibiotics against the frequently occurring bacterial superinfections were available when the “1918 influenza pandemic” took place. Indeed, penicillin—the first antibiotic in history—was discovered only in 1928 by Alexander Fleming, who, in 1945—17 years later—was awarded with the Nobel Prize by the Royal Swedish Academy of Sciences for his literally revolutionary discovery. As a consequence, the fact that no antibiotics were available by the time Spanish flu occurred, objectively makes any parallel between the two pandemics implausible, if not even impossible [6].

Notwithstanding the above, the role of antibiotic-resistant bacteria (ARB) in the clinico-pathological evolution and outcome of COVID-19 among hospitalized patients of all ages has not received, thus far, the attention such a crucial issue would deserve.

A recent report from the World Health Organization (WHO) states, in fact, that “of those taking antibiotics, 79–96% reported not having been infected with COVID-19, but were taking antibiotics inappropriately, believing they would prevent infection. Evidence indicates that up to 15% of severely affected COVID-19 patients develop bacterial co-infection and could need antibiotics, whereas 75% actually receive them” [7].

The critical relevance of this issue is well exemplified by the commonly documented involvement of ARB in severe nosocomial infections [8], as clearly shown by the 700,000 deaths caused in 2019 by ARB worldwide, 33,000 of which occurred across the European Union (EU), with 10,000 of them having been reported in Italy, the country with the highest toll in human lives inside EU due to ARB-associated/related infections [9].
